# Nanostructured Gas Sensors for Health Care: An Overview

**Published:** 2015-07-27

**Authors:** Ajeet Kaushik, Rajesh Kumar, Rahul Dev Jayant, Madhavan Nair

**Affiliations:** 1 Center for Personalized Nanomedicine, Institute of Neuroimmune Pharmacology, Department of Immunology, Herbert Wertheim College of Medicine, Florida International University, Miami, FL-33199 USA; 2 Department of Physics, Panjab University, Chandigarh-160014, India

**Keywords:** Nanotechnology, Conducting polymers, Metal oxides, Organic-Inorganic hybrid nanocomposites, Gas sensors

## Abstract

Nanostructured platforms have been utilized for fabrication of small, sensitive and reliable gas sensing devices owing to high functionality, enhanced charge transport and electro-catalytic property. As a result of globalization, rapid, sensitive and selective detection of gases in environment is essential for health care and security. Nonmaterial such as metal, metal oxides, organic polymers, and organic-inorganic hybrid nanocomposites exhibit interesting optical, electrical, magnetic and molecular properties, and hence are found potential gas sensing materials. Morphological, electrical, and optical properties of such nanostructures can be tailored via controlling the precursor concentration and synthesis conditions resulting to achieve desired sensing. This review presents applications of nano-enabling gas sensors to detect gases for environment monitoring. The recent update, challenges, and future vision for commercial applications of such sensor are also described here.

## Overview of Gas Sensor

**Figure 1:**
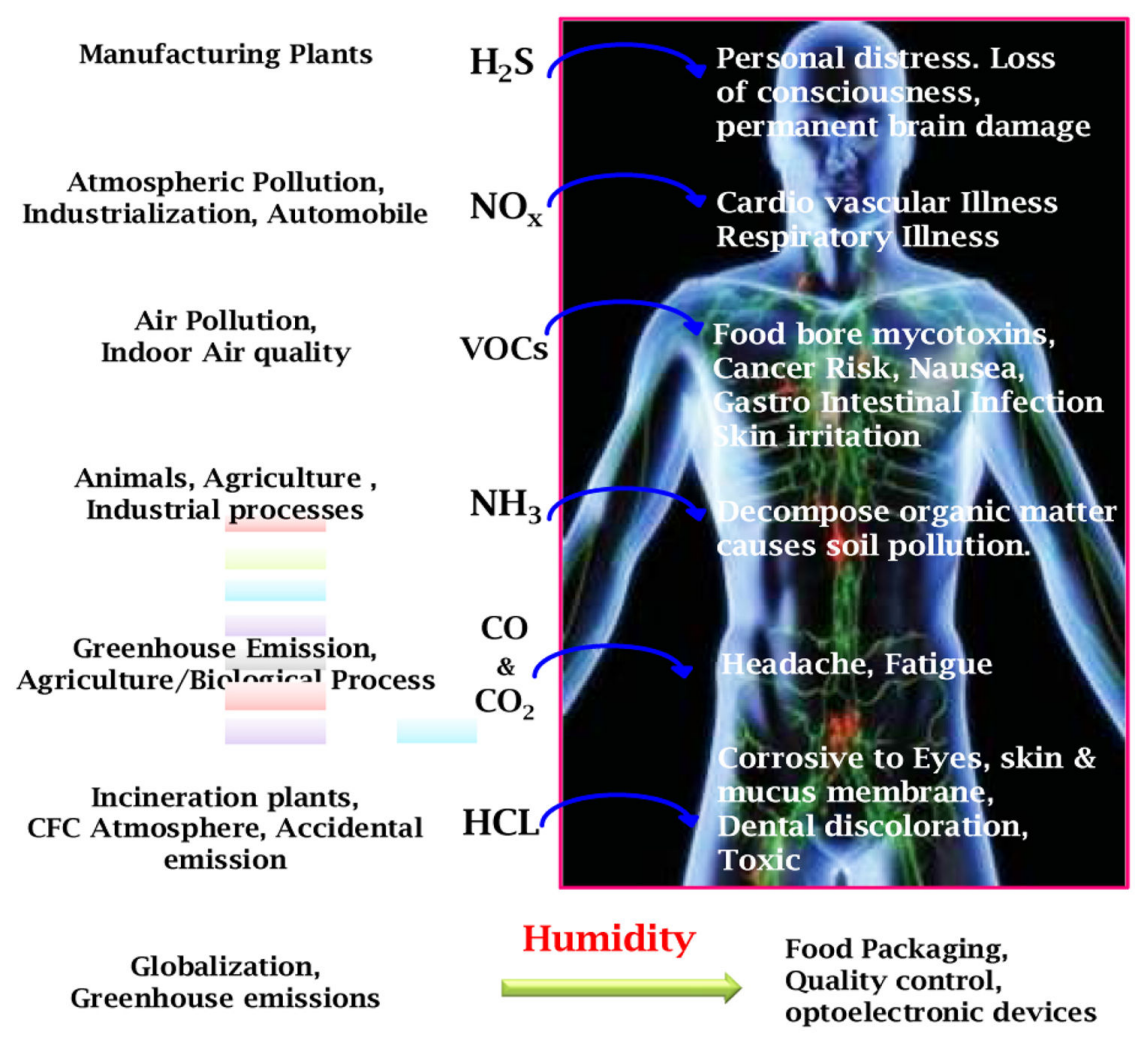
Various pollutants and possible effects.

Serious diseases due to increasing level of environmental pollution are increasing rapidly [1]. Figure 1 shows various pollutants, vapors, sources and related possible effects on human health. Thus detection of these pollutants is crucial for environmental monitoring for health care [2–5]. In this growing era, food safety [6] and environmental pollution monitoring [2–5,7,8] have become priority areas for human health care. Air pollutants originating from sources such as vehicle emissions, power plants, refineries, industrial and laboratory, include hydrogen chloride (HCl), hydrogen sulfide (H_2_S), volatile organic compounds (VOCs), ammonia (NH_3_), humidity, carbon monoxide (CO), carbon dioxide (CO_2_), oxides of nitrogen (NOx) etc., [2–5,7,8]. Soil and water contaminants can be classified as microbiological (e.g., coliform), radioactive (e.g., tritium), inorganic (e.g., arsenic), synthetic organic (e.g., pesticides), and volatile organic compounds (e.g., benzene). Furthermore, pesticides and herbicides that are applied directly to plants and soils, and incidental releases of other contaminants from spills, leaking pipes, underground storage tanks, waste dumps, and waste repositories can persist for many years and migrate through large regions of soil until they reach water resources, where they present an ecological or human-health threat.

Therefore, environmental monitoring is crucial to protect public from toxic contaminants and pathogens that can be released into air, soil, and water. In this respect, there is a considerable need to find new, user-friendly and eco-friendly techniques for the reduction and remediation of hazardous wastes such as carcinogenic organic solvents, toxic materials, and nuclear contamination which are very dangerous for environment and human health. Also a wide range of environmental problems that require solutions include global warming, ozone layer depletion, acid rain, increase in harmful waste materials in the environment, dioxin, and pollution of air and water.

**Figure 2:**
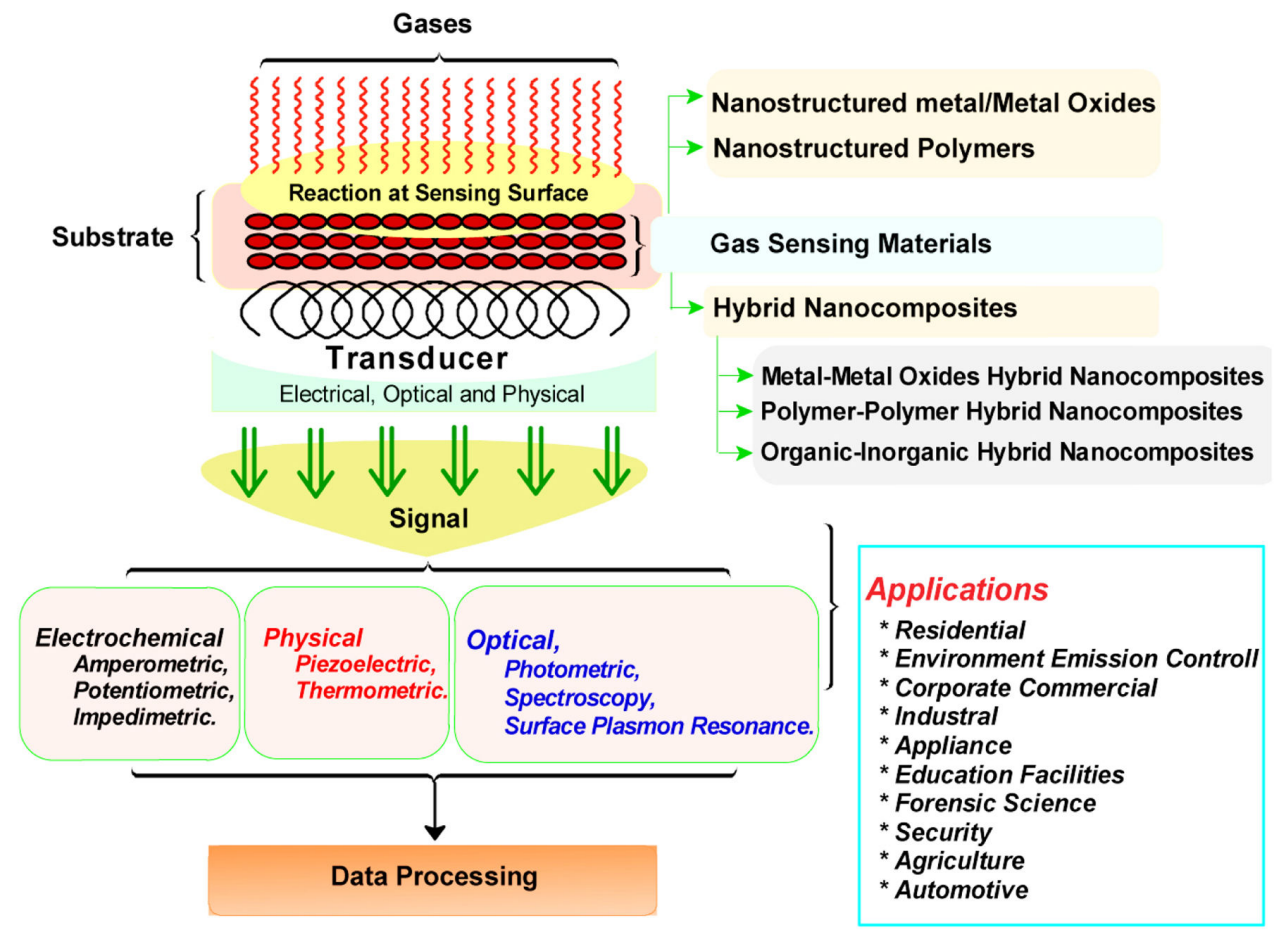
Schematic of fabrication of a gas sensor (Reprinted with permission from ref 1. Copyright 2015 ACS).

These factors resulted in continuous growth in sensor development. Fundamentally, a gas sensor is an analytical device that converts the response of a transducer to a measurable signal, when exposed to a gas. Figure 2 schematically illustrates the scheme of a gas sensor. Recently, metal oxide based gas sensors have shown great influence in many areas such as environmental monitoring, domestic safety, and public security, automotive, air conditioning for airplanes, space crafts, houses and sensors networks [9]. However, large scale applications and effective monitoring of environmental pollution require the development of cheap, small, low power consumption and reliable solid state gas sensors over the years [10–13].

Nanostructured platforms have become the materials of choice for new technological breakthrough as gas sensing application [1]. These nanostructures possess different physical, chemical and molecular properties due to the quantum confinement effect than those of their bulk component. At nanoscale, material provides high surface-to-volume ratio, which enhances their chemical activity and electron transfer kinetics resulting in signal amplification. The design of nanostructured polymers, metals, metal oxides, semiconductors and their hybrid nanocomposites are likely to pave the way for newer technologies towards chemical and physical and biological sensors. Advances surface chemistries has improved surface functionality, processibility and properties of nano-paletform to developed efficient gas/biosensors for health care [1,14–20]. The application of nanostructures as gas sensing materials has shown potential to address the challenge of low selectivity and high temperature operation in gas sensing [21] for example, nanostructured plasmonics-based gas sensors. In these sensors, the shape and surface modification of the materials, control the selective binding of analyte, leading to detection of desired targets [1,22].

Nanostructures of organic (polymers and organometallics) and inorganic (metal/metal oxides) based sensors, being accurate, are enabling advancements in environmental monitoring, domestic safety, public security, automotive technology, space applications, and sensors networks [9,23]. However, organic (conducting polymers) gas sensors have proven unstable and inorganic semiconducting mainly zinc oxide (ZnO), tin oxide (SnO_2_), titanium oxide (TiO_2_) and tungsten oxide (WO_3_) gas sensors are expensive for large-scale networking [24,25]. The utilization of organic-inorganic hybrid composite in gas sensing technology overcomes the problem of processing and stability due to the synergetic/complementary effects, which improves sensing performance.

Organic-inorganic hybrid nanocomposite is a fast growing area of research in smart advanced functional materials science [1]. Significant efforts have been focused on the ability to obtain control of the nanoscale structures via innovative synthetic approaches [26]. The properties of hybrid nanocomposite materials depend not only on the properties of their individual parents but also on their morphology and interfacial characteristics [8,27–30]. There is also the possibility of new properties that are unknown in the parent constituent materials. Efforts are continuing towards the generation of many new exciting nanocomposite materials with novel properties [26,27,31,32]. The surface modification of nanoparticles by functional monolayers of polymer “shells”, provide a means to generate composite materials with tunable surface properties (e.g. wet ability, hydrophobocity, polymerizable units, photosensitive sites or molecular recognition properties). In addition, the surface functionalization of nanoparticle allows covalent attachment, self-assembly and organization on the surfaces [8,26].

Currently, there is a strong interest in using nanosized metal or metal oxides as advanced additives in functionalization of polymers, and considerable research activities are being done in this novel field of composite science. The embedded nanoscopic metal oxide structures into the polymeric matrices represent the simplest way to take advantage of novel physical characteristics. Polymer embedded nanostructure are potentially useful for a number of technological applications, especially as advanced functional materials e.g., high energy radiation shielding materials, microwave absorbers, optical limiters, polarizers, sensors and hydrogen storage systems [33–38]. The control of nanoparticles morphology becomes an important aspect, since morphology profoundly influences the material performance. As a long term goal the development of synthesis strategies that able is to control the particle size, shape and composition independently from one another is very important, in order to allow tuning of nanocomposite properties. Depending upon the metal or metal oxide nature and polymer structure, these processes lead to the organomettalic unit incorporated into the polymer chains, metal polymer complex, metal or metal oxide clusters and nanoparticles physically connected with the polymer metrics. These nanocomposite materials have recently gained extensive interest in gas sensing application [39]. For effective monitoring of environmental pollution affordable, miniaturized, and low power solid state nanostructured hybrid composite gas sensors [10–13] with high reliability are being developed.

In brief, Nanostructures organic, inorganic, and composites demonstrate unusual optical, electrical, magnetic, and molecular properties, which may improve the sensitivity, accuracy, and stability of sensors as a function of their size and shape [40–42]. With recent advances in nanoscience and pattern recognition, sensor technology has seen major advances, allowing for the measurement of a wide range of parameters simultaneously. Along with improved sensitivity (up to 600) of the sensors, the use of nanotechnology has reduced the price of sensors to an affordable range [43–46]. Smart-single chip sensor systems with integrated nanostructures, transduction platforms and microelectronics have facilitated the fabrication of new and advanced gas sensors in a miniaturized format. These developments address the challenges of portability and have laid the foundation for wearable sensors for personalized health care [21,47,48]. Efforts are ongoing to improve the performance of gas sensors through the integration of nanostructured sensing platforms (metal oxides, conducting polymers, and composites) with desired transducer technology, device design, and materials science. These advances are also enabling applications in industries and health related products [44].

The modern topics in chemical sensors have been elucidated by Janata [49], Korotcenkov [50], Kaushik et al. [1]. Korotcenkov has highlighted that the highly potential area of sensing materials is likely to play a crucial role in the successful fabrication and implementation of sensors for desired applications [50]. The field remains an area of active research interest due to the need of reliable, miniaturized, low-cost and portable sensing systems i.e., next generation solid state sensors for environmental monitoring, food quality control, and military chemical sensors [51]. Commercialization of chemical sensors for agriculture and personalized therapeutics is also growing rapidly [52]. The development of a gas sensor is dependent on nanostructured sensing materials (as discussed above) and transduction techniques as discussed below.

## Types of Gas Sensors

**Figure 3:**
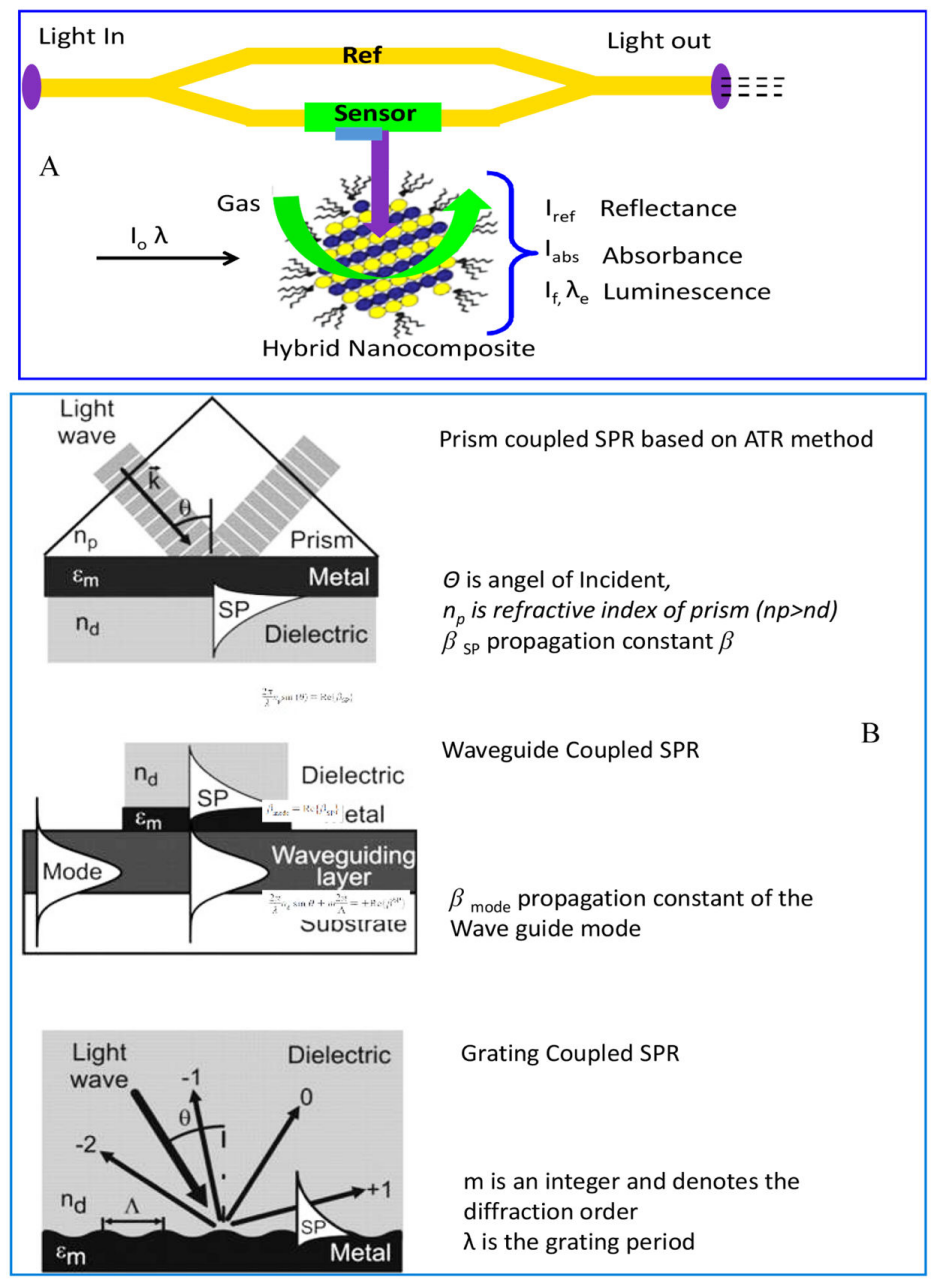
Schematic of optical gas sensor (A), and surface plasmon resonance phenomena (B).

Gas sensors can be classified according to the mode of signal acquisition such as optical (change in absorbance) [53–59], transistor and diodes (change in source-drain current at a constant potential) [60,61], piezoelectric (change in frequency) [62–65], and electrochemical including chemiresistor (change in resistance), [47] potentiometric, and amperometric gas sensor [66–68]. In optical sensors, optical transduction techniques based on absorption and luminescence have been used to yield gas analyte information (Figure 3A) [55]. Moreover, optical parameters such as refractive index and reflectivity have been explored for signal generation. Optical sensors are of two types. Firstly, direct sensors wherein, signal is generated due to intrinsic properties of sensing material such as absorption or luminescence. Photoluminescence and absorbance of surfactant aided CNT (diameter ranging from 0.9 to 1.2 nm) has been used to detect NH_3_ and NO_2_ at ppb level [69]. Secondly, reagent-mediated sensors, wherein sensing response is generated due to intermediate agent for example sensitive dye. This technique is used in a situation wherein material does not exhibit intrinsic properties.

Recently, surface plasmon resonance (SPR) sensors which referred to as excitation of surface plasmon based optical sensor are being explored for chemical and biological sensing. SPR optical sensor is a thin film refractometers sensing device which measures changes in refractive index occurring at the surface of plasmon supported metal film. On excitation by the monochromatic light, a change in the refractive index of a dielectric gives rise to a change in propagation constant of the surface plasmon (prism coupled i.e., attenuated total reflectance, waveguide coupled, and grating coupled, Figure 3B) [57,58]. The propagation constant of a radiation alters the characteristics of light wave coupled to the surface plasmon e.g., coupling angel, coupling wavelength, and intensity phase. Optical gas sensors, due to low-cost, miniaturized optoelectronic light sources and efficient detectors, have been recently utilised for multi-analyte array-based sensing [55]. H_2_S (10–100 ppm) has been detected using Cu (40 nm)-ZnO (10 nm) based SPR sensor [70]. The details of SPR sensor, technological advancements and potential application have been discussed by Homola [57,58].

**Figure 4:**
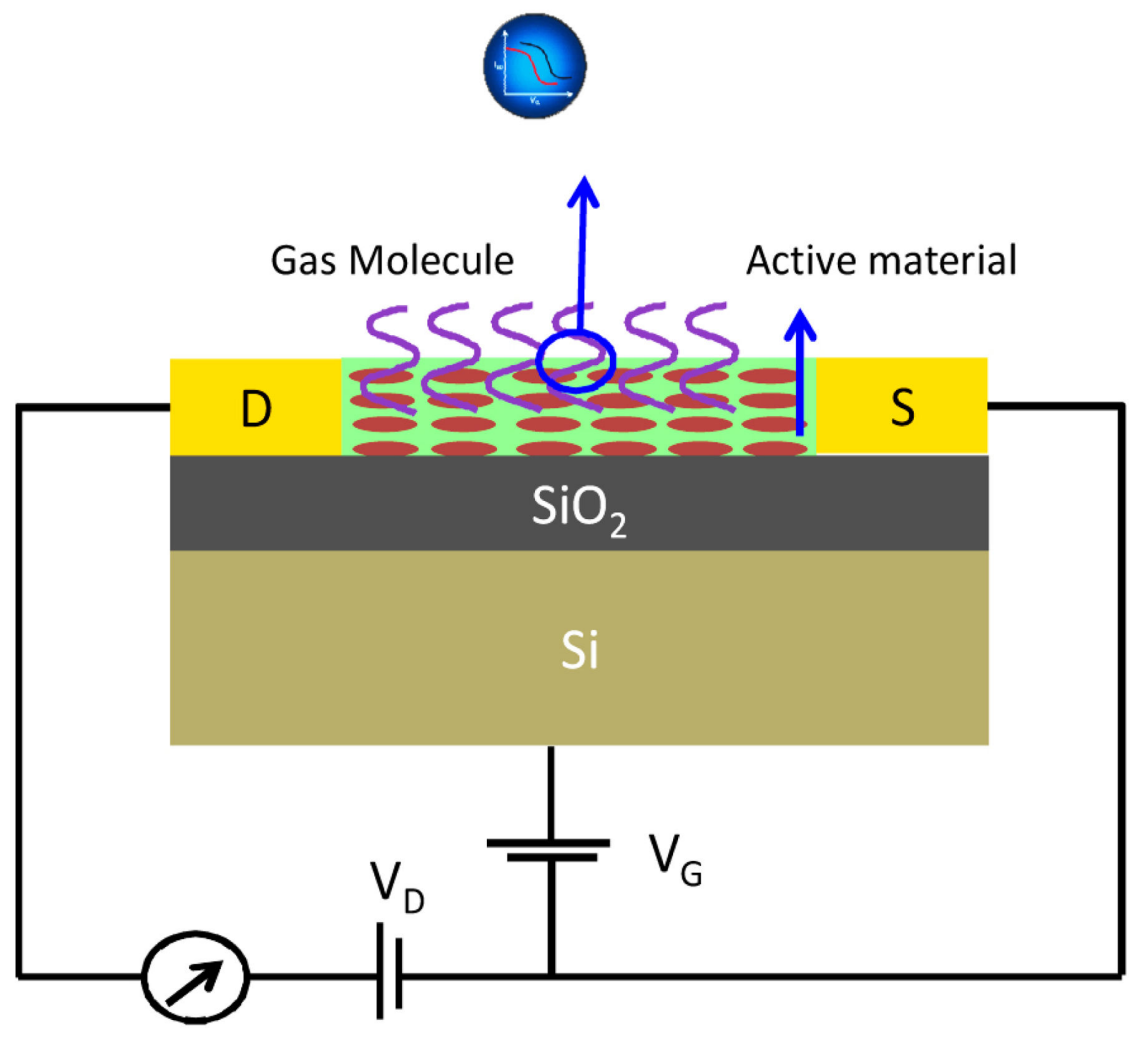
Transistor/Diode based gas sensor.

Filed effect transistor (FET) devices, due to high performance and portability have been exploited as potential chemical sensing platform [71]. These devices utilize catalytic materials as gate placed between two electrodes i.e., opposite n/p junctions (source and drain) and work as conduction channel (Figure 4). A FET based gas sensor consists of a chemical transducer, where chemical reactivity of gas molecule causes electrical change in active materials that can be monitored as a shift in the drain current (ISD), the carrier mobility and the threshold voltage of the transistor. The changes in any parameters reveal different mechanism of gas analyte transduction. The FET response is analyzed in terms of saturation current, ISD. During gas sensing, the adsorbed analyte traps the free change carrier or changes the dielectric constant of the materials which affects carrier hopping rate, leads to decrease in the charge mobility. Beside this, analyte molecule can act as a dopant cause complete or partial charge transfer in active FET materials resulting in threshold voltage (VT) change which will shift the ISD-VG curve [72]. The gas exposed FET device result in the Id, that can be estimated using $(\text{ISD})\text{sat}=W\mu \frac{C_{i}\;{\left(V_{G}-V_{T}\right)}^{2}}{2L}$

where Ci is the capacitance per unit area of an insulator, IDS is the output current in the saturation region, and W and L are the channel width and length, respectively. The curve enables deduction of the mobility √ISD= f(V_G_), which is called the field-effect mobility. V_T_ is deduced from the extrapolation of the linear region to the x-axis (V_G_=V_T_). V_T_ is deduced from the extrapolation of the linear region to the x-axis (V_G_=V_T_). Recently Mg doped InO_3_ nanohybrid based E-mode EFT sensor has been fabricated for the detection of 500 ppb CO within 4s at room temperature [73].

**Figure 5:**
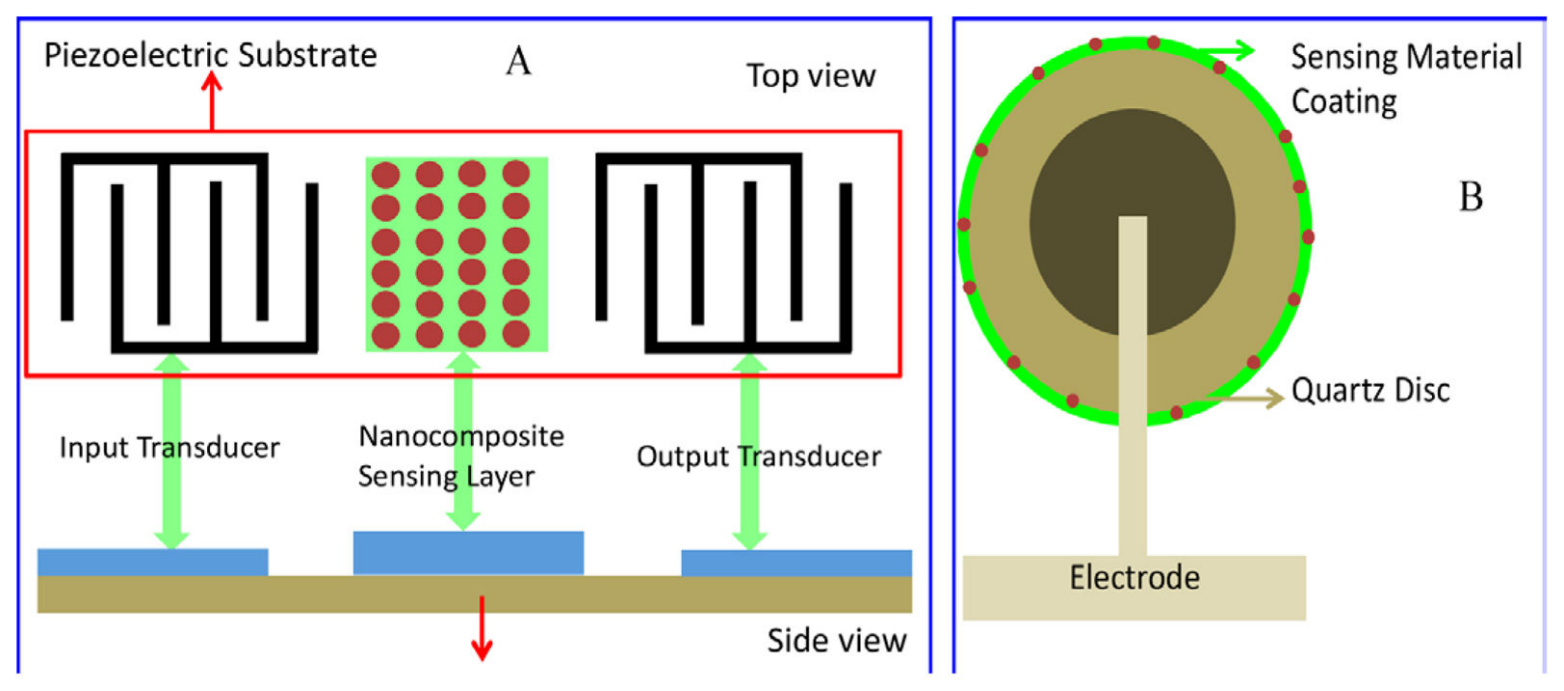
Schematic of SAW and QCM sensor.

Surface acoustic wave (SAW) device and the quartz crystal microbalance (QCM) are the two main piezoelectric gas sensing devices. A sensitive membrane is placed between an input (transmitting) and output (receiving) inter-digital transducer deposited on top of the piezoelectric substrate to fabricate a SAW gas sensor (Figure 5A). A change in mass of piezoelectric sensing coating due to gas absorption results a change in resonant frequency on gas exposure. An acoustic 2 D wave rising due to application of ac field (frequencies between 100 and 400 MHz) propagates along the surface of the crystal at a depth of one wavelength. The change in frequency due to the change in mass of gas sensing membrane on adsorption of gas can be calculated using Δf=ΔfpcvKp/Pp, where Δfp is the change in frequency caused by the membrane, cv is the vapor concentration, Kp the partition coefficient, Pp is the density of the polymer membrane used [74]. ZnO nanofilm (40 nm) coated SAW sensor has been designed to detect the liquor NH_3_ [75].

On application of ac voltage in QCM, the quartz crystal material oscillates at its resonant frequency produces the 3D wave, and traveling through the entire bulk of the crystal. On gas exposure, a change in mass of the sensing layer occurred, which alters the resonant frequency of the quartz crystal. This change in resonant frequency can be used to detect analyte concentration [74]. Figure 5B illustrates the schematic of polymer-inorganic nanocomposite based QCM gas sensing device. The sensitivity of QCM can be estimated using *f*/c = (−2.3 × 10^−6^) *f* ^2^/A, where *f* is the fundamental frequency, c is the concentration and A is the area of the sensitive film (Carey and Kowalski, 1986). It was observed that higher frequencies and smaller surface areas of sensing membrane resulting in higher sensitivity. A QCM gas sensor based on PMMA-PDLL mixed polymer was prepared to detect 80% water vapor [76].

**Figure 6:**
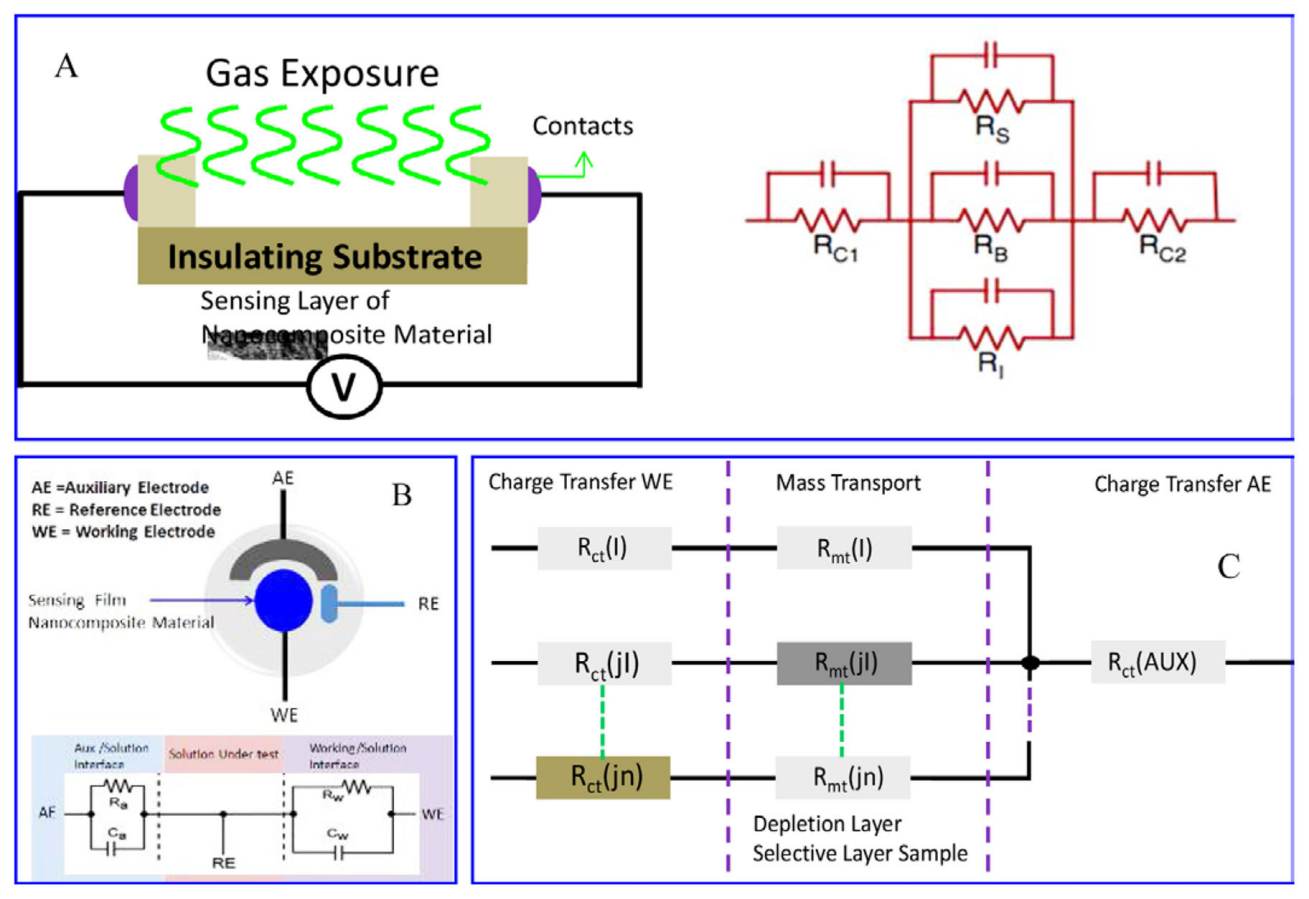
Schematic of chemiresistor gas sensor (A), potentiometric/amperometric gas sensor (B), and charge/mass transfer phenomena occurred in electrochemical reaction.

Electrochemical gas sensors (Figure 6) are electro-analytical devices which provide the information of a chemical or gases environment adjacent to an electro-active material. Depending on signal acquisition or transduction, these sensors can be classified potentiometric, amperometric, and chemoresistor gas sensing devices. Chemoresistor gas sensors measure the change in the resistance of an electrically active material such as metal oxide, polymer and organic-inorganic nanocomposite on exposure of a target gas analyte or a medium. The observed resistance change is due to absorption/adsorption of gas analytes on the electro-active charged sites of sensing materials (Figure 6A). These sensor can be cost-effective, sensitive, and can be used to study the analyte interaction with sensing materials leading to a resistance change [ΔR = (Ro−R exposure)/Ro, where Ro is resistance before exposure]. Sulfonated and ethylenediamine modified graphene based chemiresistor gas sensor has been developed to detect NO2 [77]. These sensor showed 4 to 16 time stronger response that unmodified graphene with repeatability and reproducibility [77]. Chemiresistor gas sensors based on modified interdigitated electrodes (IDE) have shown improved performance [47], due to high through-put and sensitivity.

Potentiometric sensors, thermodynamic equilibrium sensor known as ion-selective sensor (ISE) or ion-sensitive sensors (ISEs) can be utilized for monitoring of specific electrochemical reactions involving a redox reaction. These sensors provide information on ionic activity in an electrochemical reaction by measuring accumulation of a charge potential at the working electrode at zero or no current flows [78]. In potentiometric sensors, the open-circuit potential between two electrodes is monitored, which is typically proportional to the logarithm of the concentration of gas analyte, estimated using Nernst equation as

$$E=\text{Eo}+\frac{\text{RT}}{nF}\ln a$$

where *E*° is the standard electrode potential in volts, *R* is the universal gas constant (8.314472 JK^−1^ mol^−1^), T is the absolute temperature in kelvin, *F* is Faraday’s constant (9.648 × 10^4^ coulombs/mol), *n* is the number of electrons participating in the electrochemical reaction, and a=the chemical activity of the analytes. A pH glass electrode measure against carbonate-selective membrane electrode based on tweezer type carbonate ionophore has been prepared to detect CO_2_. This potentiometric sensor exhibited selective detection of CO_2_ in physiological range with a response time (t95%) 5s [79].

On gas exposure (ppb level), the chemical and diffusion processes under equilibrium conditions at the sensor surface results in a thermodynamically accurate signal. While the amperometric sensors rely on Faraday’s law and the dynamic reaction under the steady-state condition at the sensor surface (Table 1).

The amperometric sensor signal may get smaller with the size of the electrode and the rate of analyte reacting at the electrode surface, while the potentiometric sensor assumes a thermodynamic potential independent of size of the electrode. This situation becomes interesting in light of a nanostructured sensing material wherein the thermodynamic potential is characteristic of aggregates of atoms, while the amperometric sensor reaction rate is typically enhanced by the high surface areas afforded by nanostructured electrode materials.

**Figure 7:**
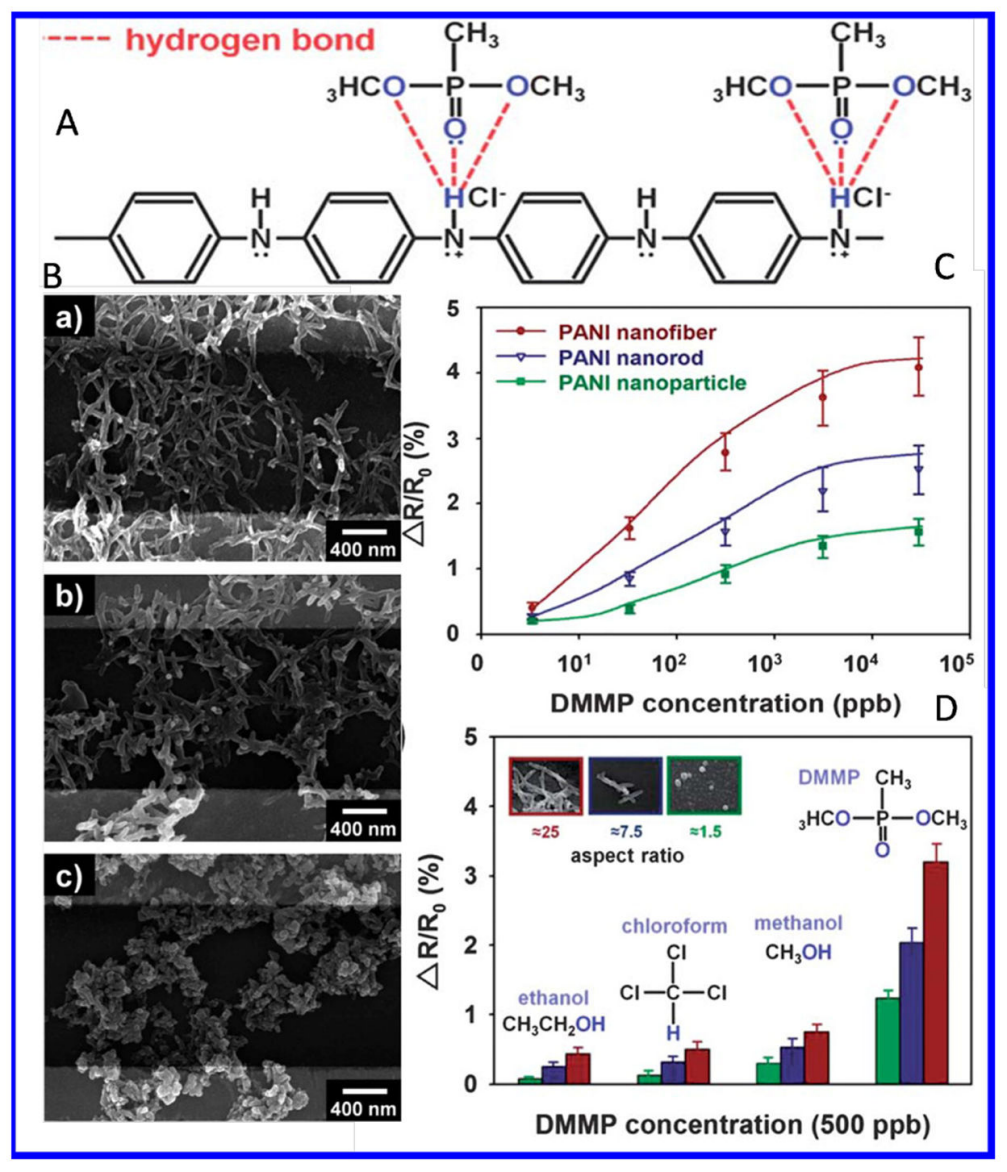
A) Proposed mechanism for hydrogen bonding between HCl-doped PANI and DMMP vapor, B) FE-SEM images of Au-IDE employing the PANI nanomaterials, C) changes in saturated sensitivity of PANI nanomaterials as a function of DMMP concentration, and (D) normalized response graphs of the PANI nanomaterials to different organic vapors at 500 ppb.[103] (Reprinted with permission from ref 1. Copyright 2013 RSC)

**Scheme 1:**
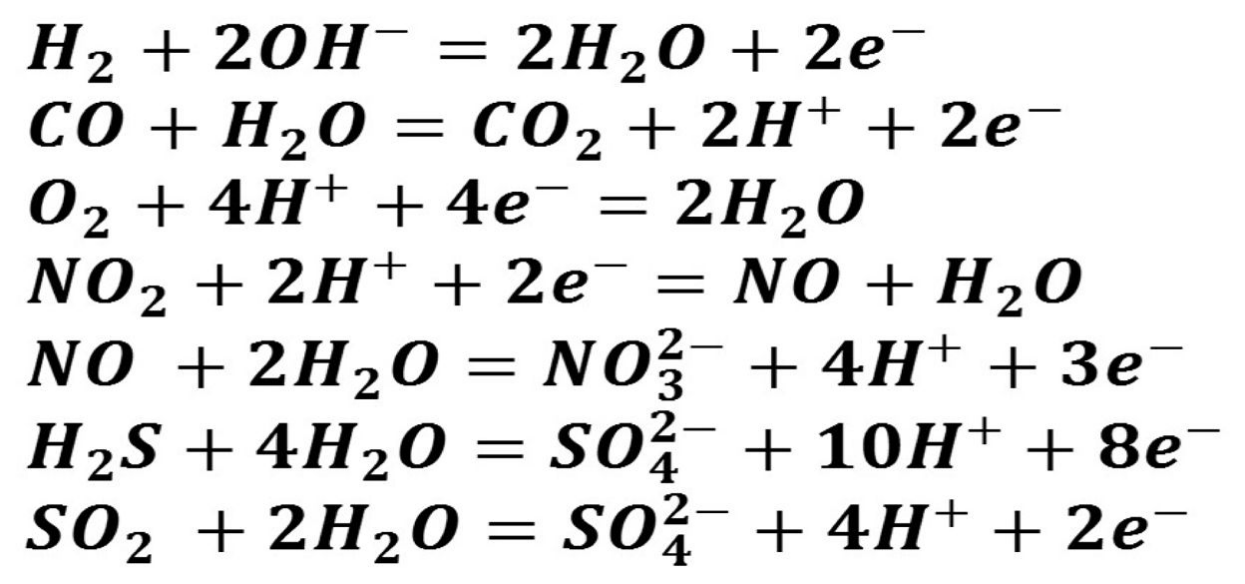
Electrochemical reaction of various gas analytes.

Amperometric gas sensor (Figure 6B) is a very important class of electrochemical gas sensor and can be used for environmental monitoring, health monitoring, homeland security, and automotive industry. These sensing devices have been used for screening and selective quantification of electro-active species in a liquid or gas phase [67]. For liquid-phase analytes, the electrodes and analytes are immersed in a common electrolyte solution. Beside this, application of amperometry to gas-phase analytes involves a unique gas/liquid/solid boundary (analyte-electrolyte-electrode) and an interfacial transport process that frequently controls the response characteristics and analytical performance. The specific design of a sensing device is crucial for the selection of an electro-active materials to enable the gaseous analyte transport towards electrode/electrolyte interface where, a fast, reversible, redox reaction can occur. This interfacial redox reaction provides the charge-transfer reaction of the analyte and the analyte’s characteristic sensor signal (Figure 7C). The selective detection of gas analyte is achieved by optimizing an electrode material to facilitate or catalyze only selected reactions or by controlling the sensing electrode potential that thermodynamically favors either oxidation or the reduction reaction for a target analyte. Nanostructures of Au, Pt, CNTs, graphene, metal oxides, conducting polymers, and organic-inorganic hybrid nanocomposites with porous morphology and high surface area have been used as sensing materials/electrode for amperometric sensing. For example, platinum oxide based amperometric sensor has been prepared for the selective detection of NO over CO. This Oxide based amperometric sensor showed a selectivity coefficients (log K_NO,j_) for sensors assembled with internal solutions at various pH values range from −0.08 at pH 2.0 to −2.06 at pH 11.7, an average sensitivities of 1.24 nA/μM and a limit of detection of <1 nM [80]. The excellent chemical stability in electrolyte solutions and high electrocatalytic activity toward analytes like H2, CO, O_2_, NO_2_, NO, H_2_S, and SO_2_ (Scheme 1) are the added advantage of these nanostructures.

## Recent Update in Nano Enabling Gas Sensors

Stetter and Li described characteristics, prospects, and advancements of amperometric gas sensors and related applications [67]. Salient features, such as low power consumption, low cost, selectivity, stability, sensitivity, and miniaturization have proven the potential of electrochemical sensors for various applications and commercial prospects. However, conventional ampereometric sensors have a slow response time. New membrane materials, sensing concepts, theoretical and modeling of integrated circuits [78] are enabling lower power consumption and improved detection limit of potentiometric gas sensors. The use of the System-on-a-chip (SoC) concept [i.e., combining technologies of complementary metal oxide semiconductor (CMOS) and microelectromechanical system (MEMS) in a sensing system that incorporates a sensor, amplifier, signal processing, analog-to-digital converter, and microcontroller on a chip] has enabled significant improvement in sensing characteristics. The SoC sensors use low power and enable reagent-less detection, that is highly reproducible with lower noise thresholds [81]. The cost of integrated electronics and poor shelf-life continue to be important in such chemical sensors [81]. Albert et al. suggested that costs could possibly be minimized using arrays of sensors to increase the feature area for multi-analyte detection via high order chemical sensing [82,83].

Another approach to classifying gas sensing devices is zero order, first order, second order and higher order. A zero order sensing device is a single sensor and is less specific, lacking the quantification of a sensing analyte. First order sensing devices are sensing arrays of identical transducers with different sensing materials e.g., high temperature metal oxide sensors. These devices are generally not very selective. The problems associated with quantification and selective detection can be addressed using second or higher order devices. These devices contain multiple transducers and arrays of sensing materials, to perform calibration using a single mixture and record the response associated with target analytes [83]. This class of sensors is currently uncommon but is important due to capability of analytes estimation, in a mixed sample [83]. Charged coupled device (CCD) chips and CMOS provide the ability to record significant data in a very short time and thus have the potential to be used in the field [84].

Introduction of networked wireless sensors is leading to new methods for chemo/bio sensing, personalized health care, and environmental monitoring. Wireless networking helps in developing low power wearable sensors that have improved reliability, and portability [85,86]. Computational methods are being used to select and optimize both the sensing materials, and surfaces, increasing the efficacy of information from the networked sensors. These computational and statistical methods are useful in estimating the magnitude of sensor-analyte interactions, and facilitate the development of new experimental methods for evaluating sensing response to target analytes [87]. As a recent development, nanostructures are being used to improve the sensing characteristics of the above mentioned gas sensors.

Organic semiconducting nanostructures offer the possibility of creating tailor-made materials, for the detection of a wide range of gases. Organic semi/conducting polymers have been found to be suitable for microelectronic devices fabrication, due to their tunable electrical characteristics [88–94]. Majority of the reports based on these polymers are focused on tailoring optical and electrical properties at nanoscale [95]. These semi/conducting polymers and other conjugated polymers have been used as the active layers in gas sensors since early 1980s [49,54,56,88]. The optical, electrical, and molecular properties of these continue to improve through synthesis of their composites and blends, ensuring improved gas sensing devices [96–98].

Nanostructured conducting polymers such as polyaniline (PANI) [99,100], polypyrrole (PPy) [101], polythiophene [102] etc., exhibit excellent sensing behavior because of their desired functionality, conductivity.

For example, nanostructures of PANI synthesized chemically at different dopant ratio (0.25, 1.0, and 2.0) onto IDE (Figure 7) [103]. The electrochemical and DMPP vapor sensing properties of PANI have been found to be dependent on PANI morphology. The nano-morphology of PANI exhibited response only to DMMP vapor (500 ppb) in comparison of ethanol, chloroform, and methanol. PANI nanowires showed higher response then PANI nanorods and nanoparticles. Figure 7 shows the interaction of PANI with gases, SEM image of nanostructured PANI, and sensing response. Recent studies have highlighted the functional properties of various conducting polymers for device fabrication [103].

However, due to the affinity of these materials towards similar moieties and moisture present in the environment, they sometimes are not selective or stable enough for gas sensing [105]. Many of the devices based on these nanostructured materials have shown poor sensitivity and slow response time because of the functional properties which are not yet fully understood [106]. Improved understanding of functional properties will provides the opportunities to synthesize new nanostructured conducting polymers that will address these issues [95,107].

**Figure 8:**
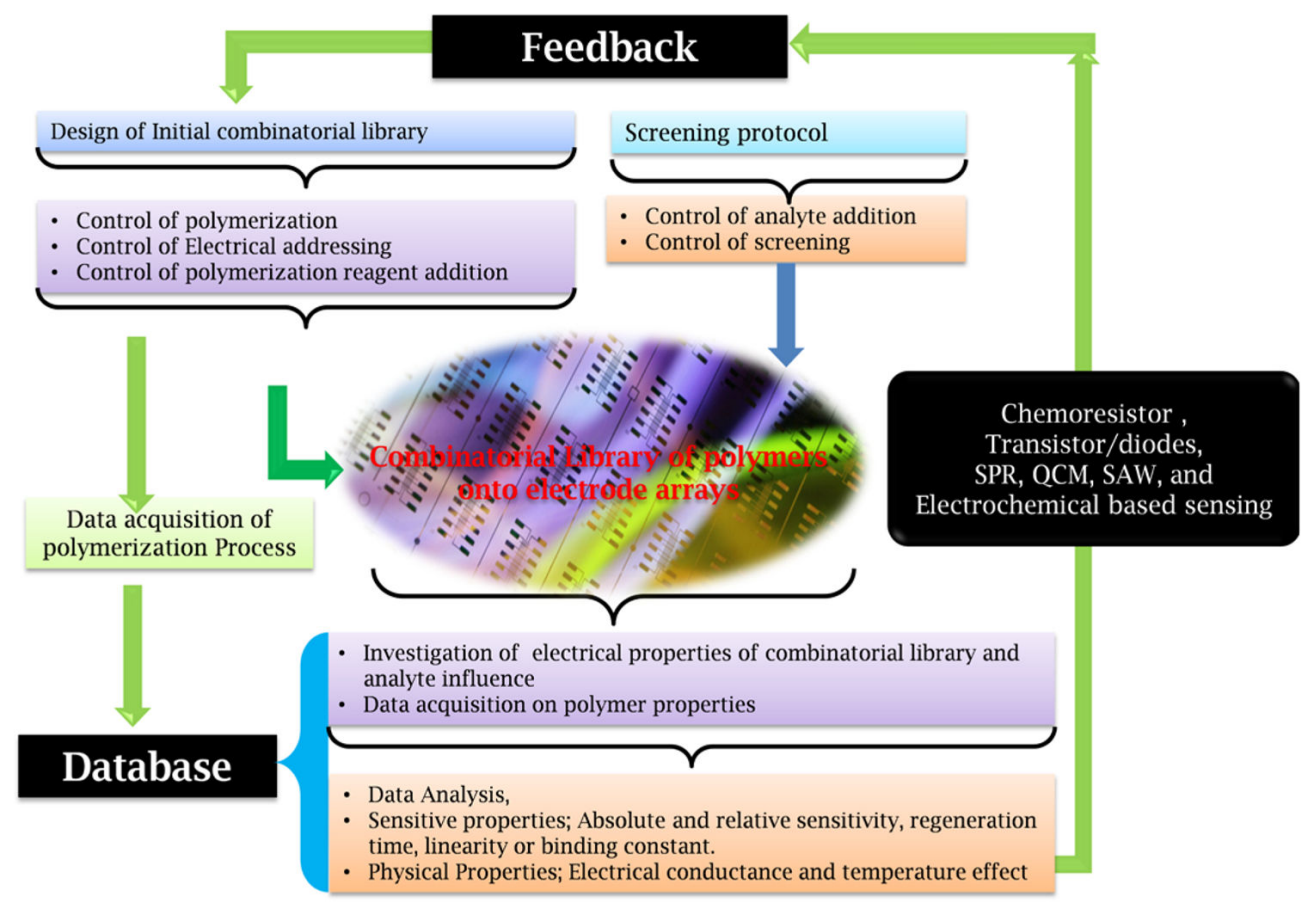
A complete system for combinatorial electro-polymerization and high throughput characterization to detect gases [104].

A combinatorial synthesis of conducting polymers which may be coupled with functional high- throughput multifunctional screening of chemosensitive properties of conducting polymers has been described by Lang et al. (Figure 8). The developed combinatorial library exhibited 100 times increase of the throughput, 4–5 times decrease of toxic waste and 1000–10,000 times decrease of possible personal exposure. This automated developed model can be used as arrays of chemical gas sensor which provide information of sensitivity, response rate, recovery rate, reversibility, reproducibility, and constant or linearity [104]

**Figure 9:**
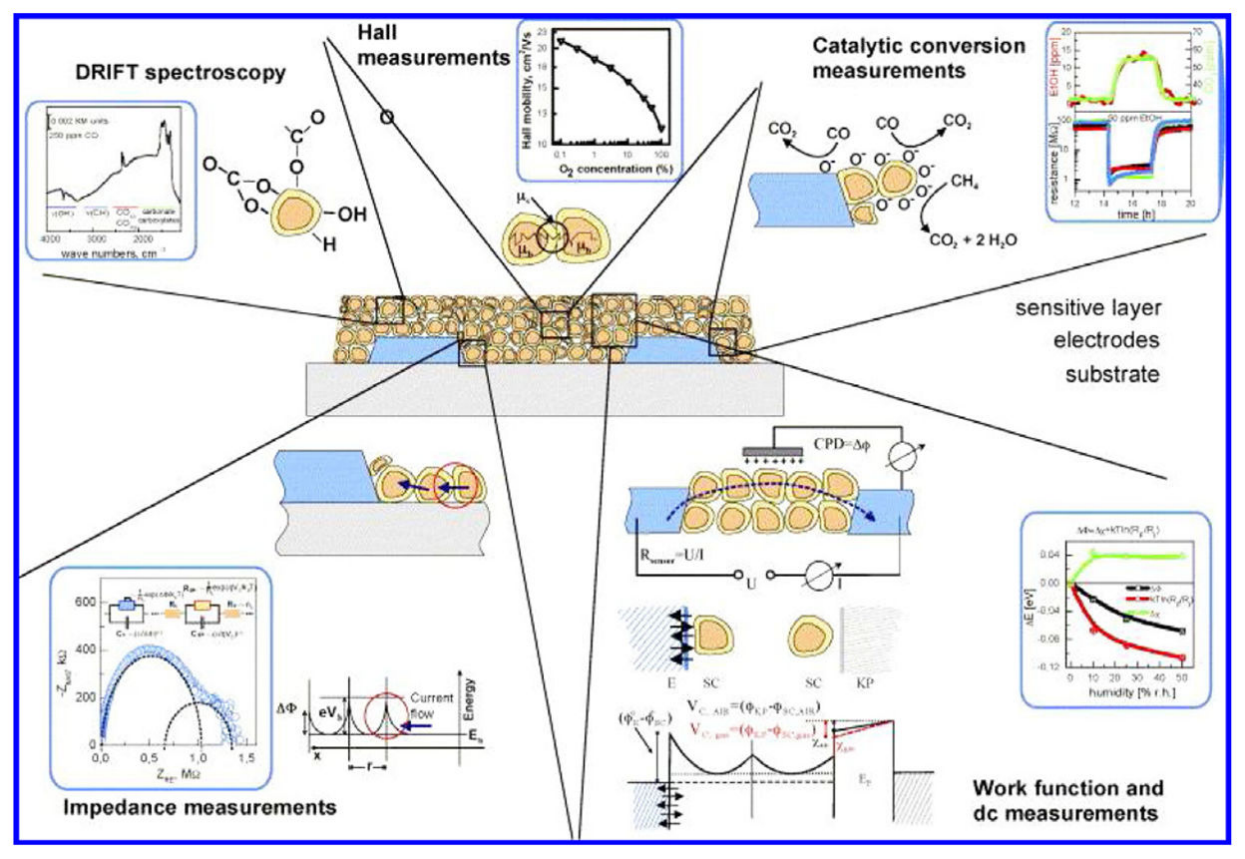
Overview of the investigated metal oxide based gas sensing methodologies.108(Reprinted with permission from ref [108]. Copyright 2007 Elsevier).

The ability of inorganic semiconductors to detect atmospheric pollutants has been extensively investigated [109–112]. Nanostructured metal oxides such as tungsten trioxide (WO_3_), zinc oxide (ZnO), tin oxide (SnO_2_), titanium oxide (TiO_2_), iron oxide (Fe_2_O_3_/Fe_3_O_4_), silica (SiO_2_) etc have been used as gas sensing materials [108,113–116]. At nanoscale, metal oxides due to changing oxygen stoichiometry possess properties that are significantly different from their coarse-grained polycrystalline counterparts, which lead to improved sensing characteristics [117]. A high degree of crystalline and atomically sharp terminations have made them very promising for a better understanding of sensing principles and for the development of a new generation of gas sensors. In these sensors, the surface effects dominate the bulk properties, because of enhanced properties such as catalytic activity and surface adsorption [108,114,117,118]. The utilization of these nano-platforms has reduced sensing instabilities caused due to drift in electrical properties [119]. Metal oxide nano-crystals such as ZnO, WO3, SnO_2_, TiO_2_, etc., can be used as resistors, in FET and optical based gas sensors [26,108,114,117,118]. The metal oxide based gas sensing techniques have been presented by Barsan et al. (Figure 9) [108].

**Figure 10:**
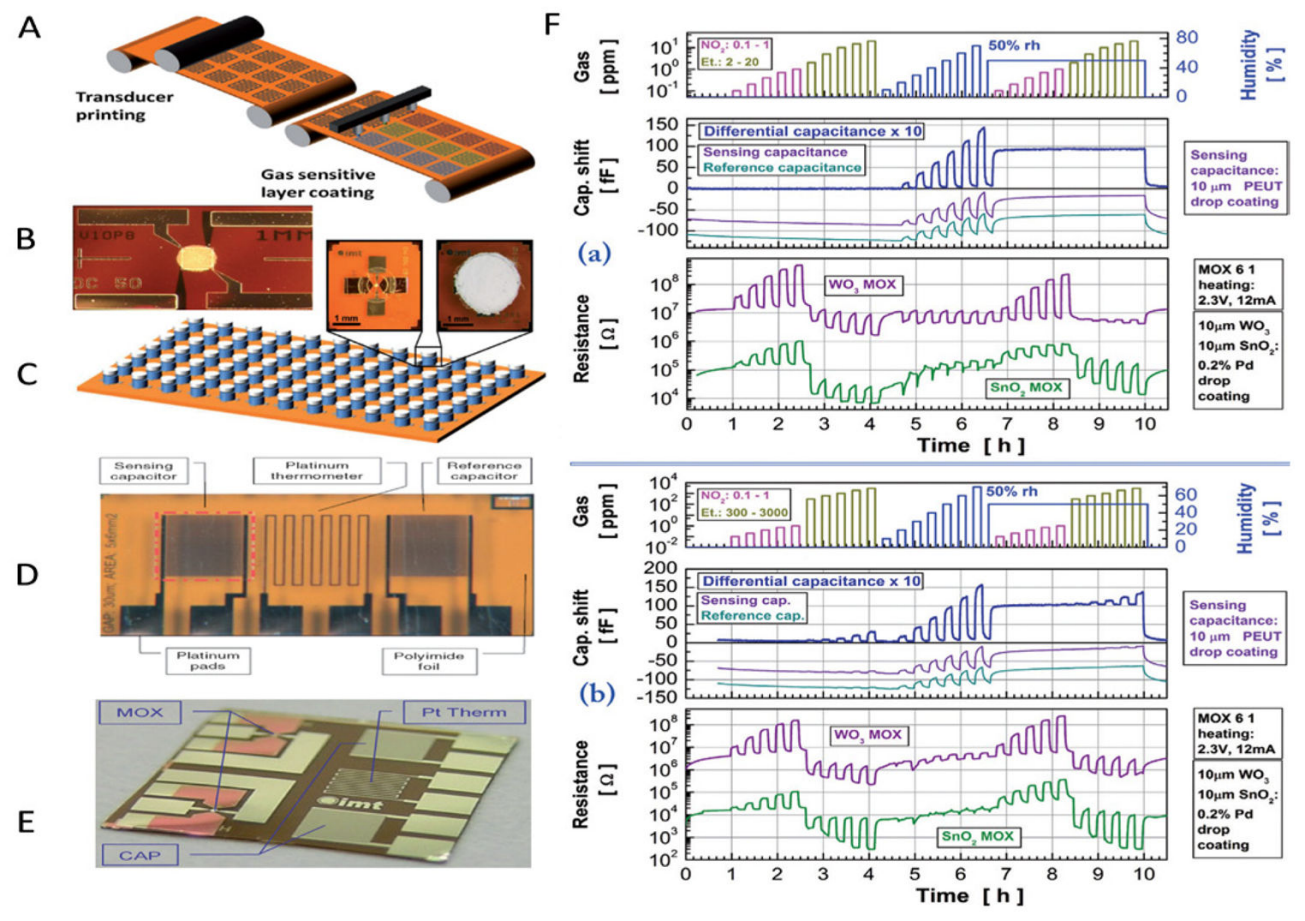
A) Schematic drawing of a roll to roll production line for chemical gas sensors on plastic foil. The transducers and coating layers are coated using additive printing techniques, such as the gravure printing of IDE and the local ink-jet printing of different sensing layers. B) Integrated metal-oxide semiconductor gas sensors on polyimide foil. C) Metal-oxide gas sensor platform with dry photoresist rims around the transducing areas and gas permeable filters. D) Integrated temperature and capacitive gas sensors on flexible polyimide foil. E) Multi-sensor platform micrograph. MOX: nanogranular SnO2 and WO3 metal oxide thick films; CAP: interdigital capacitors, one of them coated with PEUT; Pt Them: Pt -resistance thermometer. F) Multi-sensor platform signals in response to a demonstrative gas exposure protocol: (a) Low ethanol concentration range. (b) High ethanol concentration range.120 (Reprinted with permission from ref 120. Copyright 2011 Elsevier).

The ink-jet printed metal oxide nanostructured (SnO_2_ and WO_3_) based chemical sensor, fabricated on to plastic (polyamide) foil have been developed at micro-level for the detection of ethanol [120]. Integrated temperature and capacitive gas sensors on flexible polyimide foil also been fabricated for sensing of target analyte. The fabricated sensor has been tested for low and high concentration of ethanol. The step wise fabrication of flexible metals oxides based ethanol gas sensor is shown in Figure 10.

Gas sensing properties of these metal oxide nanostructures have been improved by incorporation of other nanomaterials such as carbon nanotubes (CNTs) [121,122], graphene [123], gold (Au) [124], platinum (Pt) [125], and other nanometal oxides [126,127]. CNT decorated TiO_2_ based systems have been used for gas detection at elevated temperatures. The fabricated arrays of TiO_2_-CNT systems containing [128] sensor cells has resulted in enhanced life time and accuracy of the sensors [128]. Inorganic sensors usually operate at very high temperatures (~300°C), many times leading to baseline drift and oxidation of analytes. Thus, in spite of their high sensitivity, high power consumption continues to be a serious drawback. New nanostructures have revealed good sensing properties and low power consumption in experiments, allowing for large scale manufacturing of well-organized nanostructured sensor arrays [129,130]. However, better growth control is required for commercial applications [117,118]. It has been shown that combined use of polymers and metal oxides in the nanocomposite form can help to remove their individual drawbacks, leading to an improved gas sensing devices.

It has been found that the electrical, optical, and molecular properties of organic-inorganic hybrid nanocomposites can be tailored using an appropriate synthetic route and varying precursors/operational parameters. Moreover, these nanostructures have potentially been used for various technological applications. We herein focus on the gas sensing applications for environmental monitoring. Figure 7 illustrates the organic-inorganic hybrid nanocomposites used to detect gases. The next section focuses on the gas sensing mechanism of the organic-inorganic hybrid nanocomposites.

## Organic-inorganic Hybrid Nanocomposites Based Gas Sensors

The conducting conjugated polymers, such as polythiophene (PTh), polyaniline (PANI), and polypyrrole (PPy), which have π-conjugated carbon chains, have been in focus for their gas-sensing properties [23,59]. They have many advantages over the inorganic counterparts like electrochemical reversibility, good mechanical performance, ease of preparation through chemical and electrochemical methods and operate at room temperature [131]. The polymer materials are can be useful fabricating flexible gas sensors due to their excellent mechanical characteristics [132]. On the other hand inorganic nanomaterials are more stable along with higher conductivity [133]. The gas sensors fabricated used inorganic nanostructures especially metal oxides exhibits good sensing response due to the oxygen non-stoichiometry at surface but they lack the applications due to higher operating temperature and poor mechanical properties [108,129]. Hybrid nanocomposites provide promising direction for the gas sensors development [1]. The next recent development in synthesis nanostructure and nanocomposites of precise shape and size make it possible to achieve materials with unique physical, chemical and electric properties. It was discovered that highly sophisticated factors implies for gas sensing and related to nanostructure surface can be obtained by newly developed synthesis techniques. Recently various hybrid nanocomposites consisting metal-polymers, metal-oxide-polymers or carbon nanostructured mixed with polymers have been studied for their applications as gas sensors [1]. It was observed that the incorporation of the inorganic nanostructure in polymer increases the chemical reaction activity required foe gas sensing. The enhanced chemical activity is due to the high ratio of surface atoms with free valences. The parameters such as shape and size of inorganic nanostructures, porosity, inter-phase interaction, surface and interfacial energy, catalysts activity, chemical reactivity control the response of the gas sensors. These parameters depend on the type and concentration of inorganic additives. The ratio of the organic and inorganic materials is very important and need careful optimization to achieve good sensing of a gas sensor [1].

It was also observed that the incorporation of inorganic nanostructure improves the stability of the polymer matrix e.g addition of small amount of CNTs were found to improves the mechanical properties of the polymer [127]. The incorporation of nanotubes like CNT can provide better target gas permeability for sensing material and enhances the sensitivity of the sensors.

## Opinion, Future Prospects, challenges and Conclusion

Sensors are urgently required for monitoring structural integrity of reactor containment buildings and nuclear waste repository, control of nuclear power plants, pollution monitoring and leakages of toxic gases/chemicals. The key attributes of sensors are as 1) selectivity is the most important characteristic of chemical and bio-sensors, various techniques used to impart selectivity are ion-selective electrodes, enzymes and the use of neural networks for enhancing selectivity, 2) A major requirement for sensors for use in the nuclear industry is the ability to withstand ionizing radiations and high electromagnetic interference, 3) Distributed monitoring of different parameters such as temperature, stress, neutron flux and vibration, 4) Ruggedness, corrosion resistance, useful working lifetime are the essential attributes particularly of embedded sensors and 4) Response time, sensitivity, size, cost and power consumption, are the other features which require special consideration. Recently developed gas sensors are capable of a) optical fiber technology based sensors provide distributed sensing, immunity to electromagnetic interference and resistance to damage by nuclear radiations, b) piezoelectric and SAW based devices, c) Chemical sensors based on oxide semiconducting thin films, catalytic oxidation, electrochemistry, d) 0D, 1D, 2D, and 3D nanostructures based sensors, e) Micro-cantilever and f) Single molecule sensors. Besides this, there is considerable scope to develop neural networks to provide selectivity.

Several sensors have been developed directly to generate the electronic response in digital domain and thereby avoiding intermediate signal processing. These sensors respond to shift in one of the four properties namely, i) resistivity, ii) dielectric permeability, iii) inductance and iv) emf of the physical or physico-chemical system being probed. In these sensors given physico-chemical medium is probed by placing suitable electrodes in the timing circuit of an appropriate miniature logic gate oscillator. The medium thus directly governs the timing characteristic of the oscillator. Significant progress on the development of real time remote monitoring of electrochemical parameters, pH, temperature, differential or absolute pressure level, position, absorbed radiation dose, liquid leaks, flow characterization and surface profiling etc. has been accomplished.

A fiber optic draw tower for pulling silica based optical fibers of required diameter and strength has been developed. Optical fiber based sensors for distributed temperature monitoring with sub-meter spatial resolution for the monitoring of temperature over the range of 10 to 200oC is being developed. For fabrication of thin film semiconductor and catalytic gas sensors various facilities such as vacuum deposition systems, material characterization and sensor response measurement exist. For conducting polymer based chemical and bio-sensors, both the facilities and requisite experience for preparation of Langmuir-Blodgett (LB) films and electrochemical growth of polymers exists. Preliminary experiments on conducting polymer sensors using LB grown Polycarbozole films and uric acid bio-sensors employing electrochemically grown PANI films have been carried out.

Currently, there are several activities on development of gas detectors, solid state detectors, polymer and inorganic scintillators, specialized detectors e.g. calorimeter etc. Various gas detectors that have already been developed include; ionization chambers, G. M. counters and high granularity proportional counters. Significant progress has been made in developing large area gas detectors for international high energy physics experiments. Some of the latest developments in the area of nuclear detectors are centered using high energy accelerators. Few examples include, Si-pixels detectors for ALICE (a large ion collider experiment) experiment, Si-drift and Si-strip detectors with very high position resolution for high energy physics experiments, Large area Time Projection Chamber gas detectors having large gain and very good position resolution, Multiwire Proportional Chamber, Microstrip Gas Detector, Resistive Plate Chamber and Gas Electron Multiplier based detectors, Fast and radiation hard scintillators (as Pb-Scintillator crystal) with very good energy resolution and large light output. For neutron detection combination of various phosphors and use of neutron sensitive materials are the areas of current interest.

Oxide semiconductor thin films and electrochemical sensors have been developed for various gases and chemicals. These provide sensitivity from ppm to ppb levels. Operating life of various sensors is however limited to few years. This aspect needs further investigation/development work. Significant efforts have been made to develop pattern recognition techniques for imparting selectivity to gas sensors, but they are in their infancy state and quite a good amount of work is still to be done. Optical fibres based sensors to monitor structures and operation of plants have been developed which show high resistance to radiation and are suitable for high temperature operation. Optical fibre based sensors require significant further improvements and also for monitoring of many other physical parameters and chemicals that are not presently covered. Significant progress has been reported in micro-machined silicon devices. Micro machined cantilever sensors have shown potential for detecting heavy explosive molecules like, trinitrotoluene (TNT), Dinitrotoluene (DNT), pentaerythritol tetranitrate (PETN), hexahydro-1,3,5-triazine (RDX) etc. However, integrated chemical sensors with selective response and electronics on the same chip have not yet been available.

Along with above achievements, there is a need for setting up additional facilities and expertise in a) Fabrication of large area high resolution Si-detectors possibly with industry participation, b) Large area gas detectors with high level of uniformity, efficiency and resolution, c) Detectors for pulsed neutrons in accelerators, d) Deposition of films of amorphous Si, SiC and CVD diamond etc, e) Fission detectors for neutron monitoring with improved sensitivity and those using U233 and f) Photo multiplier tubes. Additional facilities and development efforts are necessary for (a) micro-cantilever fabrication (b) 1D & 2D nanostructure based devices (c) singe molecule detection systems, (d) electrochemical gas sensors, (e) wireless and optical fibre based data communication from sensors to computerized data acquisition system, (e) Bragg and long period gratings for optical fibre sensors, (f) optical fibres with higher temperature capability, (g) neural networks.

In summary, this review explored capabilities of nano-enabeling gas sensor to environmental monitoring. The gas sensor performance dependance on electrical, optical, morphological properties of nano-sensing materials along with the on the utilized transduction method is explained in this review. Most recently explored application of organic-inorganic hybrid nanocomposites for gas sensing are also discussed here.

The state-of-the-art, challenges, future prospects of nano enabeling gas sensing for environmental moniotring is also explained. A lot of attention is yet too paid on the selection of nano-sensing materials to improve the 3S concept i.e., selectivity, sensitivity and stability for the development of gas sensing devices.

## Figures and Tables

**Figure 11:**
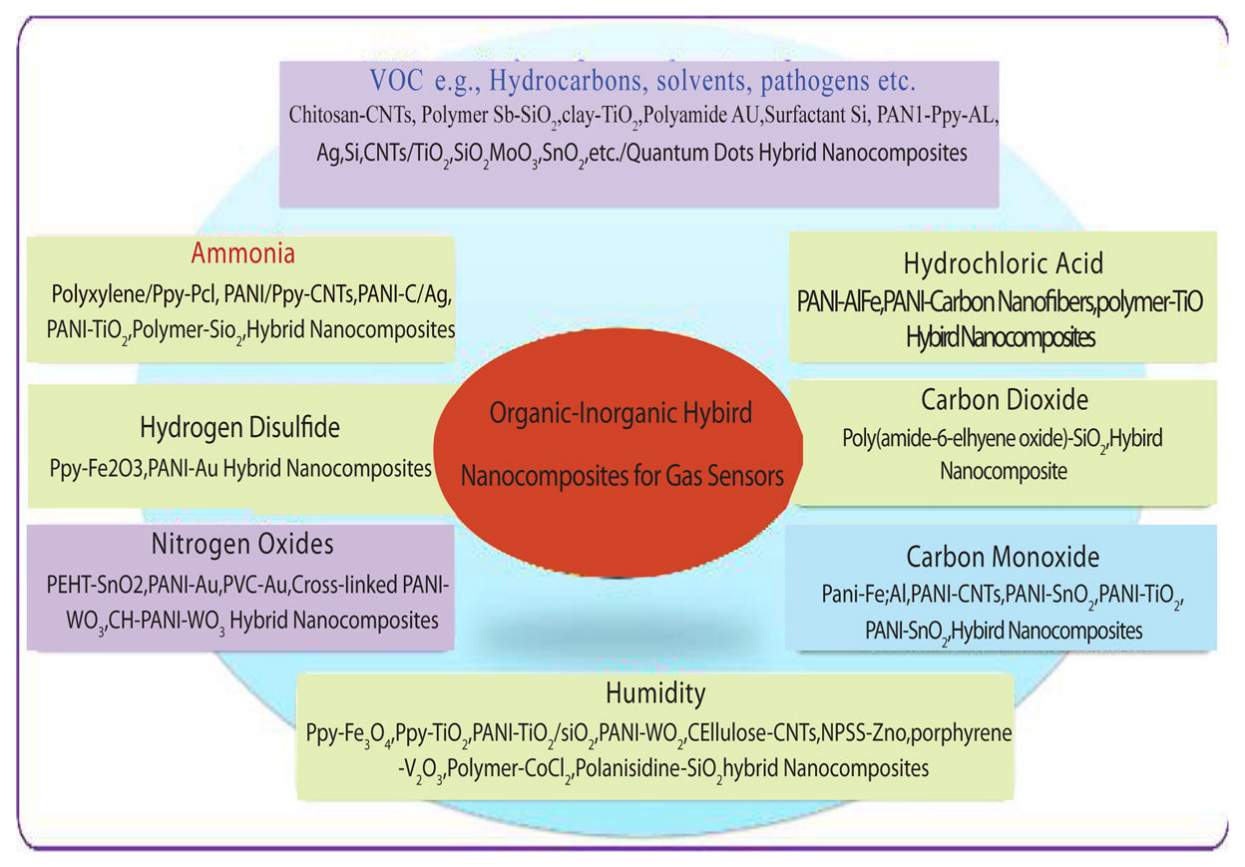
Various organic-inorganic nanocomposites for various gas sensing applications. (Reprinted with permission from ref 1. Copyright 2015 ACS)

**Figure 12:**
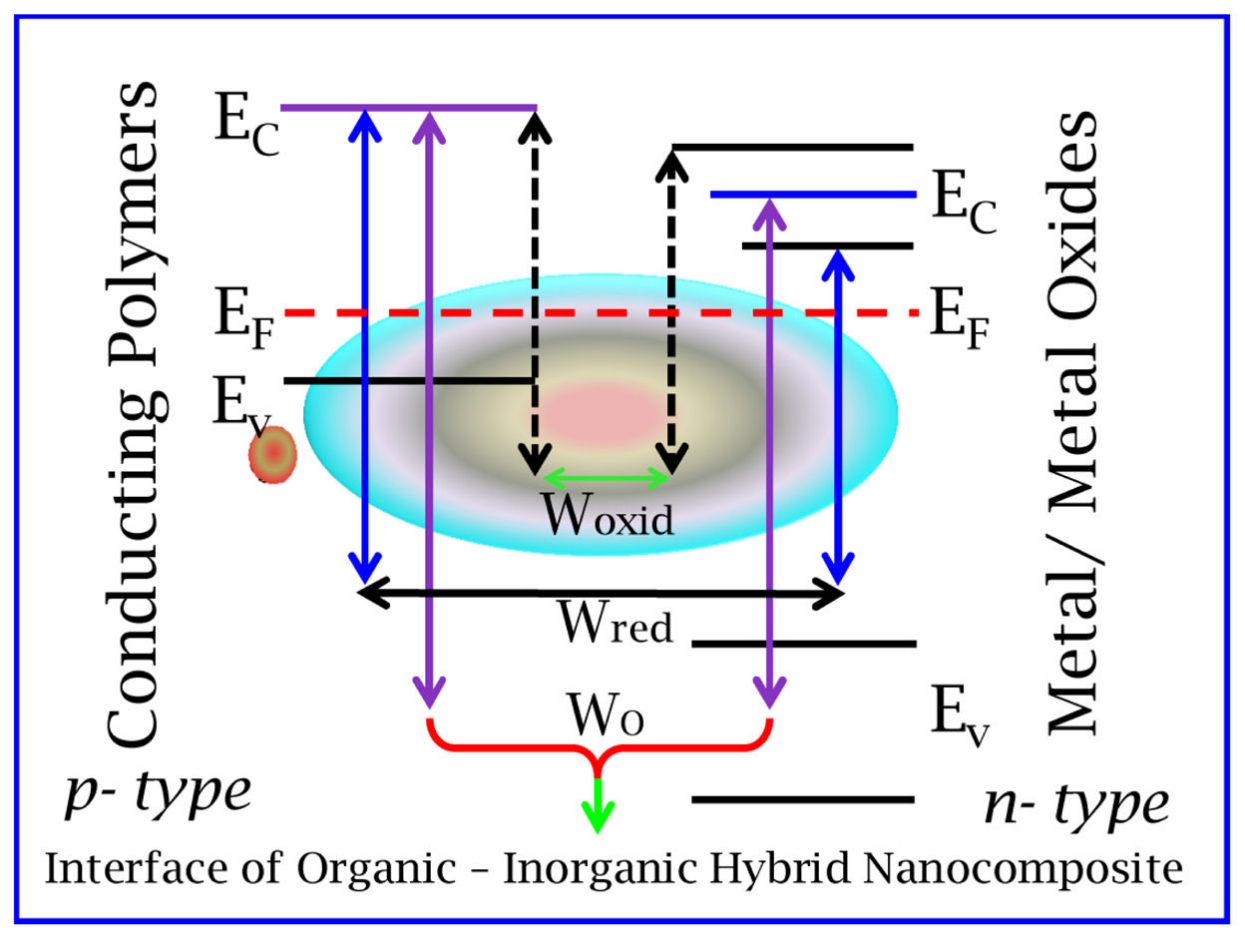
Gas sensing mechanism in organic-inorganic nanocomposite sensing systems. (Reprinted with permission from ref 1. Copyright 2015 ACS) Interface

**Table 1:** Representation of potentiometric vs amperometric gas sensor

Electrochemical Class	Sensor Signal vs [analyte]	Principle
Potentiometric	*E = kP*	Thermodynamics, Nerst Law
Amperometric	*E = Ec + klnP*	Kinetic, Faraday law
